# Learning chemistry: exploring the suitability of machine learning for the task of structure-based chemical ontology classification

**DOI:** 10.1186/s13321-021-00500-8

**Published:** 2021-03-16

**Authors:** Janna Hastings, Martin Glauer, Adel Memariani, Fabian Neuhaus, Till Mossakowski

**Affiliations:** Department of Computer Science, Otto-von-Guericke University of Magdeburg, Magdeburg, Germany

**Keywords:** Chemical ontology, Automated classification, Machine learning, LSTM

## Abstract

Chemical data is increasingly openly available in databases such as PubChem, which contains approximately 110 million compound entries as of February 2021. With the availability of data at such scale, the burden has shifted to organisation, analysis and interpretation. Chemical ontologies provide structured classifications of chemical entities that can be used for navigation and filtering of the large chemical space. ChEBI is a prominent example of a chemical ontology, widely used in life science contexts. However, ChEBI is manually maintained and as such cannot easily scale to the full scope of public chemical data. There is a need for tools that are able to automatically classify chemical data into chemical ontologies, which can be framed as a hierarchical multi-class classification problem. In this paper we evaluate machine learning approaches for this task, comparing different learning frameworks including logistic regression, decision trees and long short-term memory artificial neural networks, and different encoding approaches for the chemical structures, including cheminformatics fingerprints and character-based encoding from chemical line notation representations. We find that classical learning approaches such as logistic regression perform well with sets of relatively specific, disjoint chemical classes, while the neural network is able to handle larger sets of overlapping classes but needs more examples per class to learn from, and is not able to make a class prediction for every molecule. Future work will explore hybrid and ensemble approaches, as well as alternative network architectures including neuro-symbolic approaches.

## Introduction

In the last decades, significant progress has been made within the life sciences in bringing chemical data into the public domain in open databases such as PubChem [1]. These resources are massive in scale: as of February 2021, PubChem contains approximately *110 million* structurally distinct entries. This presents both opportunities and challenges; the annotation, interpretation and *organisation* of such huge datasets at scale becomes ever more important. Classification into meaningful groupings or *classes* enables effective downstream filtering, selection, analysis and interpretation [2]. Chemical ontologies provide structured classifications of chemical entities into hierarchically arranged and clearly defined chemical classes. To address the challenge of scale, it would be beneficial if structurally described molecular entities could be *automatically* and efficiently classified into chemical ontologies [2–4].

Machine learning has a long history of applications in computational chemistry. For example, it is used for the prediction of various chemical and biological properties from chemical structures (e.g. [5–7]). Classical multivariate statistics and machine learning approaches include logistic regression, support vector machines, decision trees and Bayesian networks. For these classical approaches, relevant features need to be specified in advance. With recent advances in algorithms, data availability and computational processing capability, multi-layer artificial neural networks, which are able to learn features directly from raw data, have begun to be used in chemistry applications [8–10].

For the purpose of machine learning, the problem of automated classification of a structurally defined molecular entity into a chemical ontology can be transformed into a multi-class prediction problem: given the molecular structure (and associated features) corresponding to a molecular entity, a model can be trained that aims to automatically predict the class or classes into which that molecular entity should be classified. The desiderata for an ontology class prediction for a molecular entity is that it should be (a) correct, i.e. it should be a class to which the molecular entity does belong; and (b) as specific as possible, i.e. there should ideally be no sub-classes of the predicted class in the ontology to which the molecule also belongs.

In this contribution, we evaluate several machine learning approaches for their applicability to the problem of classifying novel molecular entities into the ChEBI chemical ontology [11] based on their chemical structures. This is to our knowledge the first systematic and broad evaluation of machine learning for the problem of structure-based chemical ontology classification as applied to an existing ontology of the scope of ChEBI. There are challenges with the transformation of ChEBI into a form that can be used for this task, which we discuss below. We evaluate both classical machine learning approaches, which learn to predict a single “best match” class for an input molecule, and artificial neural networks, which learn to predict a likelihood of class membership for every class that the network knows about, given an input molecule. We use input encodings based on chemical fingerprints for the classical classifiers, and on the SMILES character-based line notation [12] for the artificial neural networks. The overall objective of this work is to assess how suitable machine learning is for the task of automatically predicting chemical ontology classes for novel chemical structures. We also explore whether there are performance differences between different families of machine learning approaches for this problem, and if so, whether these differences are uniform or interact with different branches of the ontology or different types of molecule.

In the next section, we present some background for our approach and discuss related work. This is followed by a section describing our methods. Thereafter, we present and discuss our results.

## Background

### Chemical ontologies

Chemical ontologies provide a standardised and shared classification of chemical entities into chemical classes. One prominent example of a chemical ontology is ChEBI [11, 13], a publicly available and manually annotated ontology, containing approximately 58,700 fully annotated entities, and over 100,000 preliminary (partially annotated) entities, as of the last release (February 2021). This includes both molecules and classes of molecules. ChEBI offers separate ontology hierarchies for the classification of molecular entities based on features of their associated chemical *structures* (atoms, bonds and overall configuration) and based on their *functions* or how they are used. For the purpose of this paper we only use the structure-based branch of the ontology.

ChEBI has been widely adopted throughout the life sciences, and can be considered the “gold standard” chemical ontology in the public domain. It has been applied for multiple purposes, including in support of the bioinformatics and systems biology of metabolism [14], biological data interpretation [15, 16], natural language processing [17], and as a chemistry component for the semantic web (e.g. [18, 19]). However, ChEBI is manually maintained, which creates an obvious bottleneck that hinders the utility of ChEBI and its chemical classification. With growth primarily limited by the manual annotation process, ChEBI is not able to scale to encompass the full scope of the publicly available chemical datasets such as are included in PubChem. Moreover, ChEBI cannot address use cases that arise in the context of novel molecular discovery, e.g. in the pharmaceutical domain where ontologies are used in the management of integrated private and public large-scale datasets as input to early drug discovery pipelines [20] for which it is important that part of the data be kept private. Moreover, it hinders applications in the context of investigations into large-scale molecular systems such as whole-genome metabolism, for which it is important that the knowledge base be as complete as possible [21].

### Automated structure-based classification in chemical ontologies

Chemical ontologies are typically implemented using logic-based semantic formalisms, including the W3C standard Web Ontology (OWL) language [22]. Based on Description Logics [23], OWL allows definitional axioms to specify necessary and sufficient conditions for class membership such that an automated reasoner can compute the hierarchy and thus detect subsumption relationships between classes automatically. Some approaches to automated chemical ontology classification have used OWL to express necessary and sufficient definitions for chemical classes in terms of atoms and bonds (e.g. [24]). However, the representation of and reasoning with molecular structures as graphs of atoms connected by bonds at the class level in OWL is hindered by the fact that class expressions in OWL must have tree-shaped models [25]. Extensions to OWL have been developed to address this challenge, including description graphs [26], description logic programs [27] and rules [28]. However, such approaches have not yet been widely adopted into tools that scale.

Alternative approaches to automate structure-based chemical ontology classification have harnessed highly optimised cheminformatics algorithmic approaches for graph substructure and superstructure matching, in combination with weakly axiomatised ontologies. One of the first such attempts was “CO” [29], an ontology of  260 classes based on combinations of chemical functional groups generated with the cheminformatics “checkmol” software. This was later developed into the more complete approach reported in [4] which used a custom general molecular fragmentation algorithm to enumerate all the parts of each molecular entity and assert those as axioms in a resulting OWL ontology to make the parts available for ontology reasoning about the class hierarchy. However, this strategy quickly creates a combinatorial explosion of content, which becomes inefficient as the size of the knowledge base grows.

Within the cheminformatics domain, the SMARTS language has been developed to encode general structural features of chemical classes [30]. SMARTS allows the specification of structures of molecules and allows specifying generalised attributes of atoms and bonds in particular locations within the overall graph structure and overall molecular features such as the total number of rings. In addition, SMARTS allows composition by means of the standard logical operators *and*, *or* and *not*. SMARTS has been used for structure-based chemical ontology classification: first OntoChem’s SODIAC [31] commercially, and then the ClassyFire application non-commercially [3] used the approach of associating chemical ontology classes with SMARTS patterns for the purpose of embedding cheminformatics knowledge within a chemical ontology, and offered accompanying software packages that are able to use this information to perform automatic structure-based classification of chemical entities in chemical ontologies. At the time of writing, the ClassyFire algorithm is the state of the art for a structure-based chemical ontology supported by automated classification, in terms of size (9000 definition rules, and an associated ontology of 4825 classes) and adoption. However, ClassyFire is based on rules rather than adaptive learning technologies, thus, updating the integrated knowledge system can only be accomplished by updating the software and its associated rules.

### Machine learning for chemical classification

In cheminformatics, machine learning approaches are commonly used for the prediction of function from structure, e.g. for the prediction of bioactivity classes, for virtual screening, or for the prediction of physicochemical properties [32]. They have recently also been applied for chemical class prediction: In [33], a back-propagating artificial neural network is applied to classify natural products. This classifier–named NPClassifier–is trained on a dataset of around 73,000 natural products sourced from public databases including Pubchem, ChEBI, and the Universal Natural Products Database. The classification structure which these molecules were organised into had just three hierarchical levels: 7 *Pathways*, 70 *Superclasses* (each of which is classified into a *pathway*), and 653 *Classes* (each of which is classified into a *superclass*). NPClassifier uses chemical fingerprints as input encoding. The fingerprint they used was a version of the Morgan fingerprint modified to integer format to include counts of atoms and substructures, calculated with RDKit [34]. After evaluating which would work better (single task or multi-task models), they used three single-task models–one model for each of the classification hierarhichal levels. They report that NPClassifier outperformed ClassyFire for a selection of overlapping classes at the superclass and pathway level (1000 members), and in particular performed well on polyketides and lignans. In the evaluation at the class level (100 members), NPClassifier outperformed ClassyFire for 50 out of the 62 classes chosen for the test. However, this task uses an artificially restricted classification problem in the sense that they only accommodate three levels of hierarchy, while in the general problem of classification in chemical ontologies, classes can be arranged in a hierarchy of arbitrary depth.

In another recent publication, machine learning was used to predict class membership directly from mass spectrometry features in an untargeted metabolomics study [35]. This is an important use case, as in untargeted metabolomics there are often many features which relate to ‘unknown’ molecular entities and thus are not mapped to defined molecular entities about which metabolic information is known; however, they may nevertheless share detectable chemical classes. In this effort, the chemical fingerprint was used as an intermediary structural representation for learning purposes: one network was trained to predict chemical fingerprints from mass spectrometry features, and another to predict class membership from fingerprints.

In another recent application learning in chemistry, a recurrent neural network was trained on SMILES strings as grammatical structures in order to predictively generate novel molecular entities that could be considered ‘grammatically correct’ [36], structures that were valid. The generated molecules showed evidence of being sampled from the same problem space as the original training molecules, thus, were good candidates for being novel molecules with a similar bioactivity profile. While this work does not try to predict classes for molecules, it uses an encoder for SMILES strings that is similar to the one we use.

## Methods

### Preparation of ChEBI data for learning

In order to prepare the ChEBI ontology for the learning task, we downloaded and parsed the OBO format export of ChEBI’s ontology using Python’s PRONTO library [37]. We use only the hierarchical (*is a*) relationships from the ontology for this study.

ChEBI’s ‘chemical entity’ branch of the ontology includes both fully-defined molecular entities with associated molecular structures as well as chemical classes that group together multiple molecular entities. Although the molecular entities are often leaves in the ontology, from ChEBI’s perspective these are all classes, and indeed in some cases there are hierarchical relationships defined between molecular entities with structures, such as between *alanine* (CHEBI:16449) and *L-alanine* (CHEBI:16977). However, for the purpose of this learning exercise we need to introduce a distinction between *classes*, which we define as those classes within ChEBI that subsume multiple members, and *members*, which we define as those classes in ChEBI that (a) are leaves of the ontology hierarchy and (b) are associated with a fully-defined molecular structure, indicated by the presence of an associated SMILES annotation. By this distinction, *alanine* is a class with *L-alanine* as a member.

The number of classes within the full ChEBI ontology, their unbalanced sizes, and the problem of multiple inheritance at every level, makes it challenging to train classifiers on the whole ontology, in particular simple classifiers that predict just one class for a given chemical structure as input. We thus implemented a dedicated selection strategy that does not use the full ChEBI ontology, but rather chooses classes, and sub-samples randomly from their members, such that the result is *balanced* (i.e. each class having an identical number of members). This provides a more well-defined task for the classification algorithms. We restrict the sampling of classes to those that are classified beneath the ‘molecular entity’ root of ChEBI, as this is where the bulk of the leaf members with defined molecular structures are found.

**Fig. 1:**
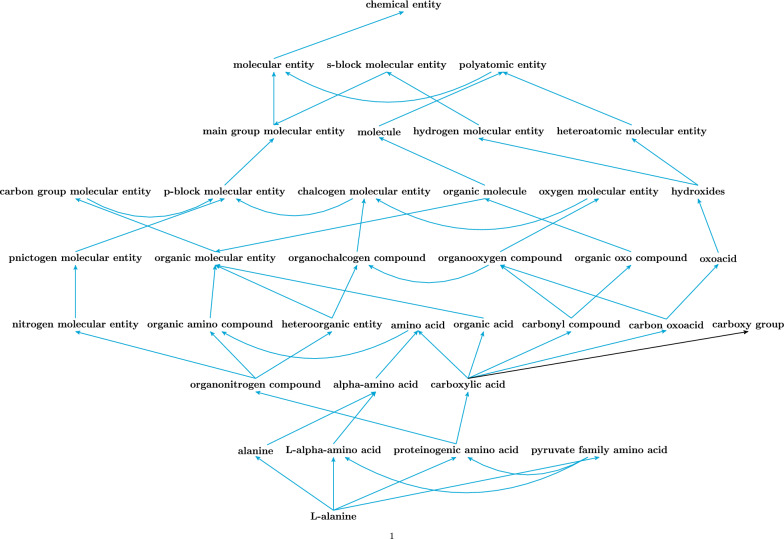
The figure illustrates the many branching and partially overlapping ancestor classes subsuming the molecule *L-alanine* (CHEBI:16977). Each of the illustrated mid-level classes similarly contains an overlapping range of molecular entity leaf members. For this reason, the ChEBI ‘chemical entity’ ontology can be described as *diamond-shaped*. Blue arrows indicate subsumption relationships and the black arrow indicates a parthood relationship

Alongside the need to prepare a balanced dataset in terms of the number of members per class, it is also important that the members with structures are selected so that individual members are not duplicated across multiple classes, in order to enable the clean separation of the dataset into training, test and validation sets. However, in practice the ontology contains a large percentage of overlapping members between classes, since the ontology classes higher up in the hierarchy describe general chemical features that in many cases can be compositionally combined in classes lower down in the hierarchy [2], as illustrated in Fig. 1. To mitigate this challenge, the selection process only sampled each leaf member structure once, assigning it as a member of the training data for just one class, even though in the actual underlying ontology that molecule in fact belongs to multiple classes. This is an artificially restriction for the purpose of the learning task: we sub-sample the leaf members with molecular structures for each class such that no leaf member with structure is selected for more than one class.

Sub-sampling members for classes such that no classes have any shared members can potentially introduce a bias. In order to perform the selection in a way that will have the least impact by minimising the discrepancy from the actual ontology structure, the classes were first sorted from the smallest to the largest (in terms of the number of leaf members with structures) so as to prioritise classes with fewer members over classes with more members, and thus reduce the amount of sub-sampling required. Following this strategy, we iteratively selected sets of *N* classes with *M* randomly sampled member structures, where *N* and *M* were specified dynamically, so as to be able to evaluate performance across a range of different problem sizes. A dataset containing *N* classes of which each has *M* members will be denoted as $N \times M$. No additional chemical prioritisation strategy (e.g. to ensure chemical diversity in selected members) was used in the selection.

### Input encodings

We used three different strategies to encode the molecular structures of individual molecules. First, chemical fingerprints were calculated for each structure using the RDKit [34] software library’s RDKFingerprint, represented as a bit string with size 1024 bits. The RDK fingerprint represents a hash of random walks of length 1–*n* (we used the default, *n* = 7) through the molecule including atom types, bond types and branches, thus represents a generalised representation of the substructures present in the molecule. Note that in this work we did not consider alternative fingerprints. For comparison, the natural products classifier mentioned in the related work [33] used an enhanced version of the Morgan fingerprint in which a count of distinct occurrences of substructures was embedded into the fingerprint vector (not just presence or absence).

**Fig. 2:**
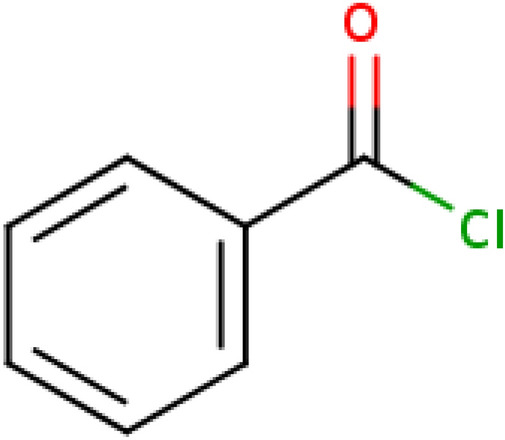
CHEBI:82275 benzoyl chloride, ClC(=O)C1=CC=CC=C1

For learning systems that require fixed-length inputs, as do many of the classifiers that we tested, fingerprints are a feature-rich input encoding that has regularly been used. However, encoding structural features via fingerprints may lose crucial information concerning the actual arrangement of these features. Artificial neural networks are able to exploit variable-length inputs. SMILES can be regarded as a language with atoms and their bonds as the alphabet. Figure 2 illustrates the molecular structure and SMILES of a representative ChEBI entity. In connection with the learning objectives for our classification problem, language models have been shown to have the potential to be applicable for chemical classification [38]. Thus, we also explored using the full SMILES representation. Similar encoding approaches have been successfully employed in analyses of natural language [39]. These approaches have shown promising results with a tokenization on the character level.

**Fig. 3:**
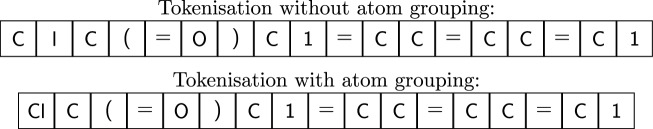
Character-wise tokenisation with and without atom groupings

In comparison to linguistic examples, SMILES strings present information more compactly, without the equivalent of word boundaries. Despite their similar abbreviations, Hydrogen (H) and Helium (He) or Phosphorus (P) and Lead (Pb) have very different properties. Therefore, it may be beneficial to remove the burden of learning these differences from the dataset by using an encoding that encodes atoms as units. For this reason we used two alternative encodings: one where each token represents a character of the SMILES string, and one where letter combinations that represent an atom are encoded as one token. This approach is depicted in Fig. 3. Note the different handling of Chlorine (Cl) in the two approaches. Multi-digit cycle pointers are still encoded as sequences of digits, as they may become arbitrarily large, which does not work well with the token embedding used in our networks.

### Classifiers used

We perform a broad evaluation of learning approaches that have shown good results in the field of data science. We reasoned that trying a wider range of different approaches may give deeper insights into the structure and properties of the problem than just trying a single approach. We implemented the classifiers using Python’s SciKit-learn library, and the artificial neural networks using Python’s TensorFlow library.

The following subsections briefly describe the different algorithms for the different classifiers. For all algorithms except the artificial neural networks, fingerprints with fixed input size are used as the input data. Only the artificial neural networks are capable of processing SMILES, which have variable length.

#### Logistic regression

Classifiers based on logistic regression represent the classification problem as a relationship between the categorical dependent variable (i.e. ontology class membership) and the independent variables (i.e. the molecular structural features) by estimating probabilities using a logistic function, which is the cumulative distribution function of the logistic distribution. An individual logistic regression function can discriminate between two classes, thus to scale to multiple classes we have used the “one-vs-rest" strategy in which a separate classifier is trained for each node.

#### K-nearest neighbor

The popular K-nearest neighbor approach classifies a new data point by selecting those *k* existing data points that are the closest to the new data point w.r.t some metric in a multivariate space. The labels of this selection are used to determine the class of the given data point. This is done either by a simple “majority vote" or more sophisticated methods based on the distances between the points.

#### Decision tree

Decision trees are a supervised learning method which fits a model that predicts the categorical dependent variable (i.e. class membership) by learning simple decision rules inferred from the input data features. The primary advantage of decision trees is that they can be interrogated and visualised; explanations (in terms of rules) can be given for their outcomes. A disadvantage is that decision trees are susceptible to overfitting and to bias in unbalanced datasets.

#### Random forest

Individual decision trees are prone to over-fitting. Random forests aim to overcome this by training multiple trees on random subsets of the training set to induce different tree structures, then, using an ensemble approach to derive the correct prediction. The correct estimation is expected to be present more often in the results, and the individual fitted tree structures are different enough to allow more precise predictions.

#### Naive Bayes

Naive Bayes methods are supervised learning approaches based on applying Bayes’ theorem with the “naive” assumption of conditional independence between features. Naive Bayes approaches have the advantage of requiring less training data than other approaches and showing good performance in many real-world situations despite this somewhat unrealistic assumption.

#### Linear discriminant analysis

One of the main drawbacks of Naive Bayes methods is their independence assumption. Linear Discriminant Analysis follows a similar mechanism, but does account for possible co-variances among features. The estimated probability distributions are still expected to follow a normal distribution, which leads to poor performances for classes that depend on different combinations of features.

#### Support vector machine

Support vector machines are supervised learning approaches that map the example data as points in a high-dimensional space such that the examples of the different categories are divided by a gap that is as wide as possible. While by default support vector machines perform linear separation, they can be used for non-linear classification using what is known as a ‘kernel trick’: a non-linear kernel ‘reshapes’ the problem space to make it more easily separable by linear functions. In this work, we use three different kernels:

Linear: A linear kernel aims to separate the problem space using a linear plane–similar to linear regression–but adds additional penalties for points that are too close to the line of separation. This results in a more robust classification.
RBF: A radial basis kernel reshapes the problem space according to the euclidean distance to some centroid. This makes points that are close to the center of the centroid more easily separable from those that are not.
Sigmoid: A sigmoid kernel reshapes the problem space with respect to a sigmoid function (e.g. tanh). This makes points on the high end of the function more easily separable from the ones on the low side.

#### Artificial neural networks

Character encodings from molecular line notations produce a sequence of vectors that represent the structure of a molecule. Such sequences of vectors are common in natural language processing, for which different types of gated networks have been successfully applied [39, 40]. The most prominent gated networks are Gated Recurrent Units (GRU, [41]) and Long short-term memories (LSTM, [42]). Whilst GRUs show better performance when it comes to runtime, the controllable memory functions of LSTMs improve the in-process information management. This kind of management is especially important for molecules, because information that may belong to the same cycle of atoms may be separated far apart due to the way cycles are broken up during the SMILES generation. Given these properties, we decided to use LSTMs.

**Fig. 4:**
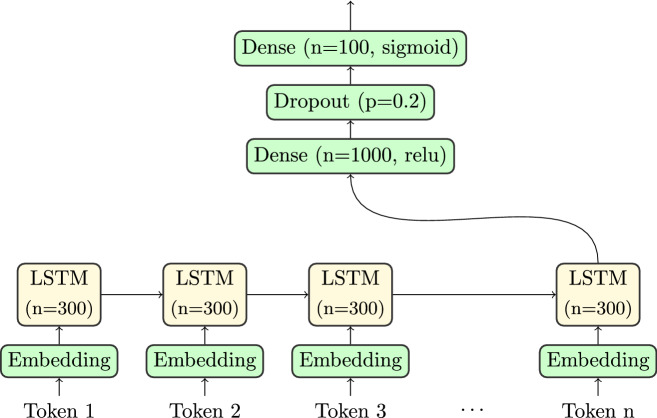
One-directional LSTM

Figure 4 illustrates the way we apply LSTMs to the different tokenisations. Each token is embedded into a real vector. The LSTM consumes each of these embeddings in sequence and adapts its internal activation and memory state. The output of the last cell is then passed though a dense layer with a trailing dropout to prevent overfitting. The results are passed to a dense layer with sigmoid activation function. The result is a vector of values between 0 and 1, representing membership values of each class.

### Evaluation

Each approach has been trained on a series of *N*x*M* datasets for different problem sizes, in each of which *N* is the number of classes, and *M* is the number of class members for each class, generated from the CHEBI ontology as described in the Methods. It is important to bear in mind that different classification approaches may have different goals which approximate the overall objective of structure-based chemical classification in different ways. For the logistic regression classifier, a one-vs-rest classifier is built for each class in the problem set in order to determine the best matching class for a given input. In the remaining classical approaches, a multi-class single-label classifier directly outputs a single predicted best match amongst the classes in the problem space. For the LSTM, the network predicts the likelihood of membership of multiple classes simultaneously, which are then subject to a threshold-based selection to determine predicted membership of classes.

Additionally, for the LSTM, the resulting dataset for each problem size has been split three ways, into a train, test and validation set. The network was trained on the training set, while the validation was used to evaluate the hyper-parameter configuration. The test set has only been used for the final evaluation. For the other classification approaches which do not have hyper-parameters to configure, the dataset has been split two ways into a training and a test set.

For each problem size, class and classification approach, we obtain a set of class predictions for the test set of molecular structures (either encoded as fingerprints or directly into the network). These predictions are scored against the classes that they were sampled from according to the usual metrics of *precision*, *recall* and *F1*, where precision is defined as $TP / (TP + FP)$ and recall is defined as $TP / (TP + FN)$ (TP = true positive, FP = false positive, FN = false negative). The F1 score is the harmonic mean of the precision and the recall, i.e. $2*precision * recall / (precision + recall)$.

We perform one additional evaluation, in which we calculate, for each predicted class or classes for a given input molecule, how far that prediction is from the "ground truth" of the asserted parents of that molecule in ChEBI. For each predicted classification, and for each asserted parent, we calculate a distance based on the path length, i.e. the number of subclass relationships that must be traversed to get from the predicted parent to the actual parent. If the predicted parent is an actual asserted parent, this length is 0. If the predicted parent is the parent of the asserted parent, the length is 1 and so on. Note that by virtue of our selection strategy, not all the classifications we input to the training and prediction exercise are direct asserted parents. With this distance-based metric we are able to compare not only our approaches, but also the state of the art approach, ClassyFire.

Note that we only evaluate performance in terms of metrics for prediction correctness; we do not evaluate the temporal aspect of performance in terms of the time taken to perform the classification task.

## Results and discussion

We explore our results in several sub-sections that each focus on a different aspect of the comparison. First, we look at the results by problem size. Thereafter, we compare the different algorithmic approaches to learning. We then interpret the best and worst predictions, and finally we compare the predictions to the state of the art tool.

### Evaluation results by problem size

We have used different sizes of classes and members selected from ChEBI, in order to evaluate how the performance of classification scales with the size of the problem. Note that we do not include the LSTMs for every one of the problem sizes, as in general training artificial neural networks is more expensive than other classifiers, and such networks require larger training sets to operate.

There are two different relevant dimensions for “size of the problem": one is the size of the ontology, which we proxy with the number of classes selected for the classification task, and the other is the size (in number of leaf member compounds with structures) of each class within the ontology, which we proxy by selecting different numbers of leaf members with structures from the selected classes. In general, we would expect that a smaller number of classes, and a larger set of examples to learn from, would yield an easier task for most automated approaches to classification.

**Fig. 5:**
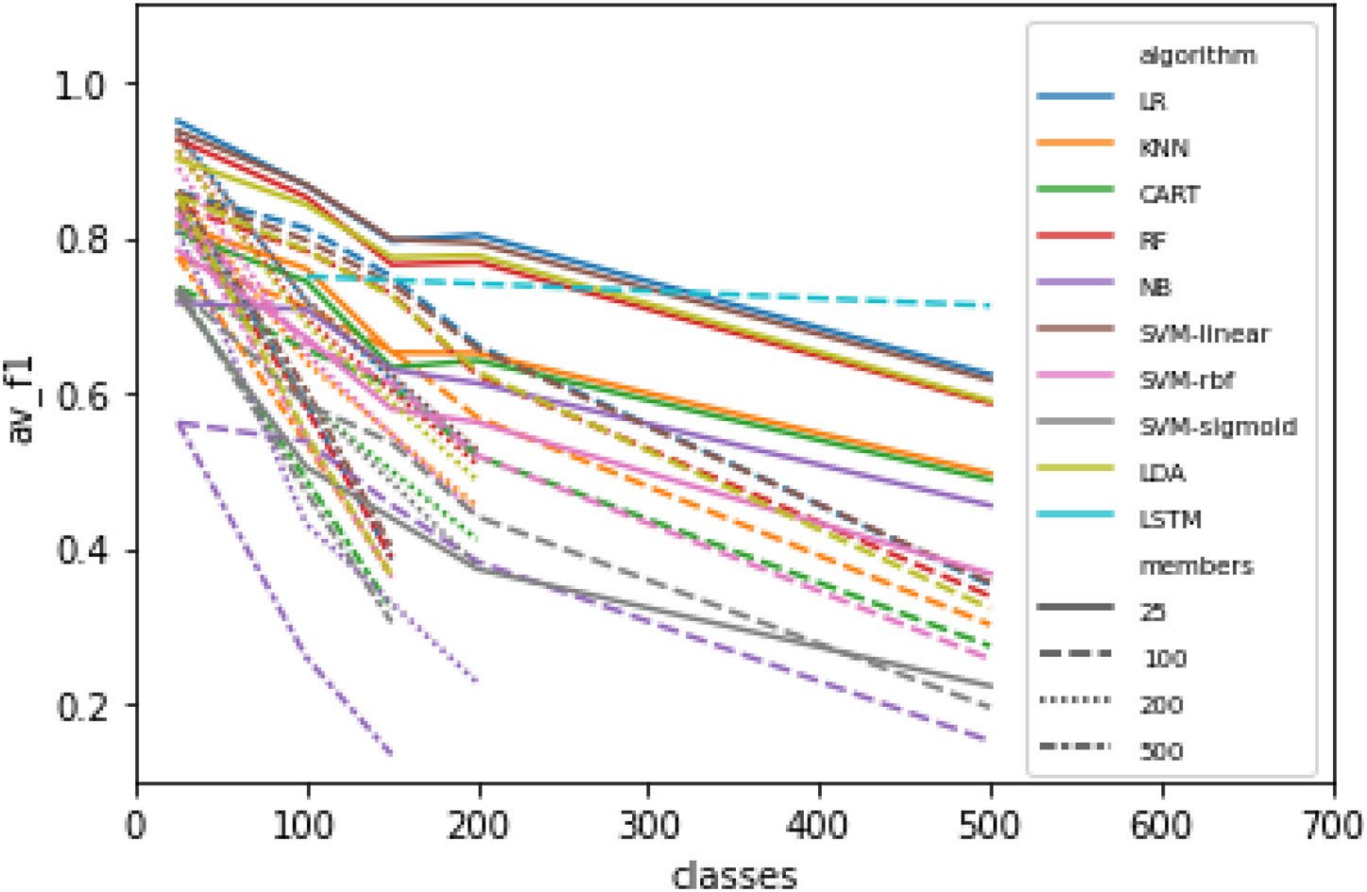
Mean F1 score across all classes for different problem sizes. LR = logistic regression;KNN = K-nearest neighbours; CART = decision tree; RF = random forest; NB = naive bayes; SVM = support vector machine; LDA = linear discriminant analysis; LSTM = long short-term memory network

**Fig. 6:**
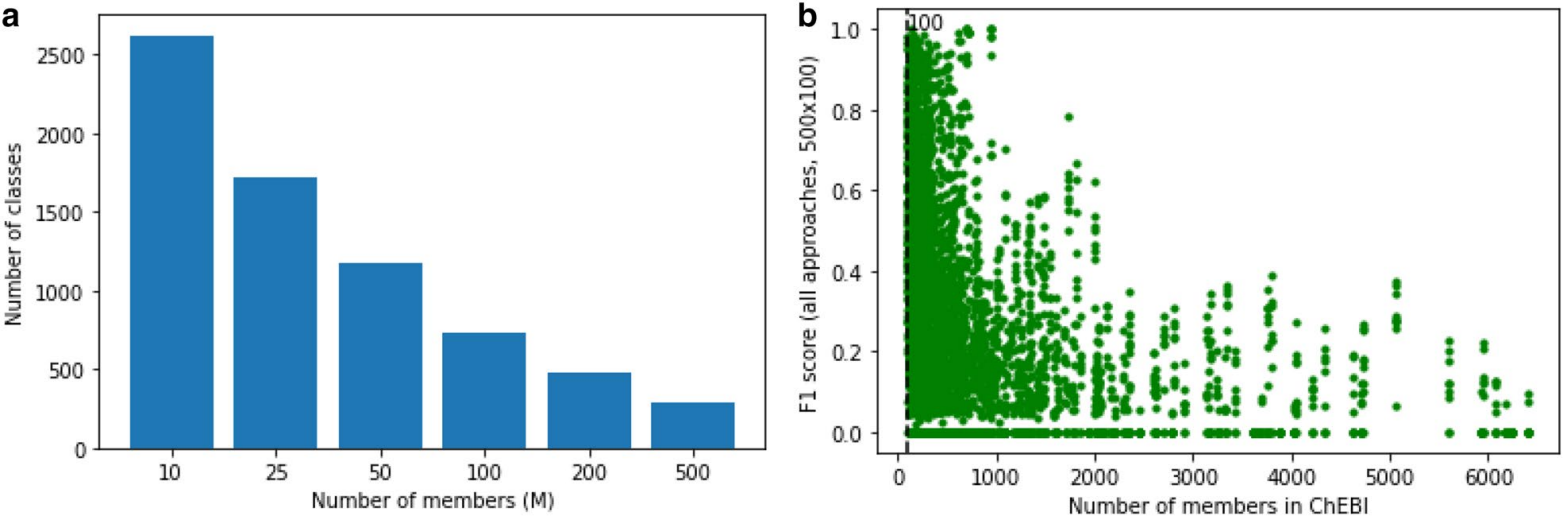
**a** Number of classes with at least *M* members, for different sizes of *M*, in ChEBI. **b** Performance of all models in the 500x100 problem for classes with different sizes. Each dot represents the F1 score for a single class in a single model

As expected, we find better scores in general for problems involving a smaller numbers of classes. As Fig. 5 depicts, there is a robust general relationship between the number of classes given to the problem, and the average F1 score for predictions of members of those classes. For some of the classifiers, 25 classes had worse performance than 100 classes, but from 100 to 500 classes there is a robust effect of decreased performance across all classifiers. Although expected, this result nevertheless suggests a challenge for the translation of these results into a classifier that scales to the size of the full ontology: there are currently 1721 classes in ChEBI that have at least 25 members (Fig. 6a), and this number can only be expected to grow.

On the other hand, perhaps contrary to what might be expected, *smaller* numbers of members per class appears to result in *better* performance for this problem. In Fig. 5, different line styles indicate different numbers of members selected per class. With 500 members per class, performance is worse than with 100 members per class, and performance with 25 members per class is best of all.

While perhaps a surprising result, this is likely an artifact of the class selection strategy coupled with the partially overlapping nature of classes within the chemistry domain: sampling larger numbers of members per class forces the sampler to use classes that *have* larger numbers of (leaf) members, which in turn means that the classes will be broader and have fewer uniquely defining features. While our training data was created in such a way that each leaf member is sampled just for one class, nevertheless, each molecule in practice belongs to many different classes, and by definition many of the classes overlap in terms of their membership. And indeed, this is what we observe: Fig. 6b shows the different F1 scores for classes across all the different models for the 500x100 problem size, in the context of the actual class size in the underlying ontology in terms of the number of members. There is a robust decrease in performance with the increase in class size.

This presents a challenge in particular if the problem of structure-based ontology classification in chemistry is framed as a multi-class, single-label prediction classification problem, which it is for the classical learning approaches that we evaluated. We therefore also explored whether multi-label hierarchical classification approaches could mitigate the shortcomings of the classical classifier algorithms applied to this problem. Any of the above classifiers can be used together with a hierarchical classification strategy [43]. In a hierarchical classifier, hierarchical relationships between the target classes are taken into consideration by training separate classifiers for each of the higher-level nodes in the hierarchy, which then derive predictions just for the levels beneath them, in a chain of nested classifiers that are iteratively applied until a leaf node is reached. This is also closely related to the approach that was taken in the natural products classifier mentioned above [33], as in that work a different classifier was trained for each of their three hierarchical levels. We thus evaluated a hierarchical classification approach based on subsets of ChEBI corresponding to the hierarchy above a given set of selected classes. However, we found that this approach in practice did not scale to subsets of ChEBI classes at the problem sizes we have used, likely because the need to extract a spanning sub-graph in which all classes are connected to the root to apply the hierarchical approach generates large graphs with significant multiple inheritance even for smaller problem sizes. Moreover, performing hierarchical classification in the case of ChEBI classes would involve significant redundancy because the classes at the intermediary levels have so much mutual overlap in terms of their lower-level members. Artificial neural network-based approaches can learn hierarchical structures directly, as we will see, thus, we did not further explore hierarchical classifiers at this stage, although we may return to this in future work.

### Comparison of different algorithmic approaches

**Fig. 7:**
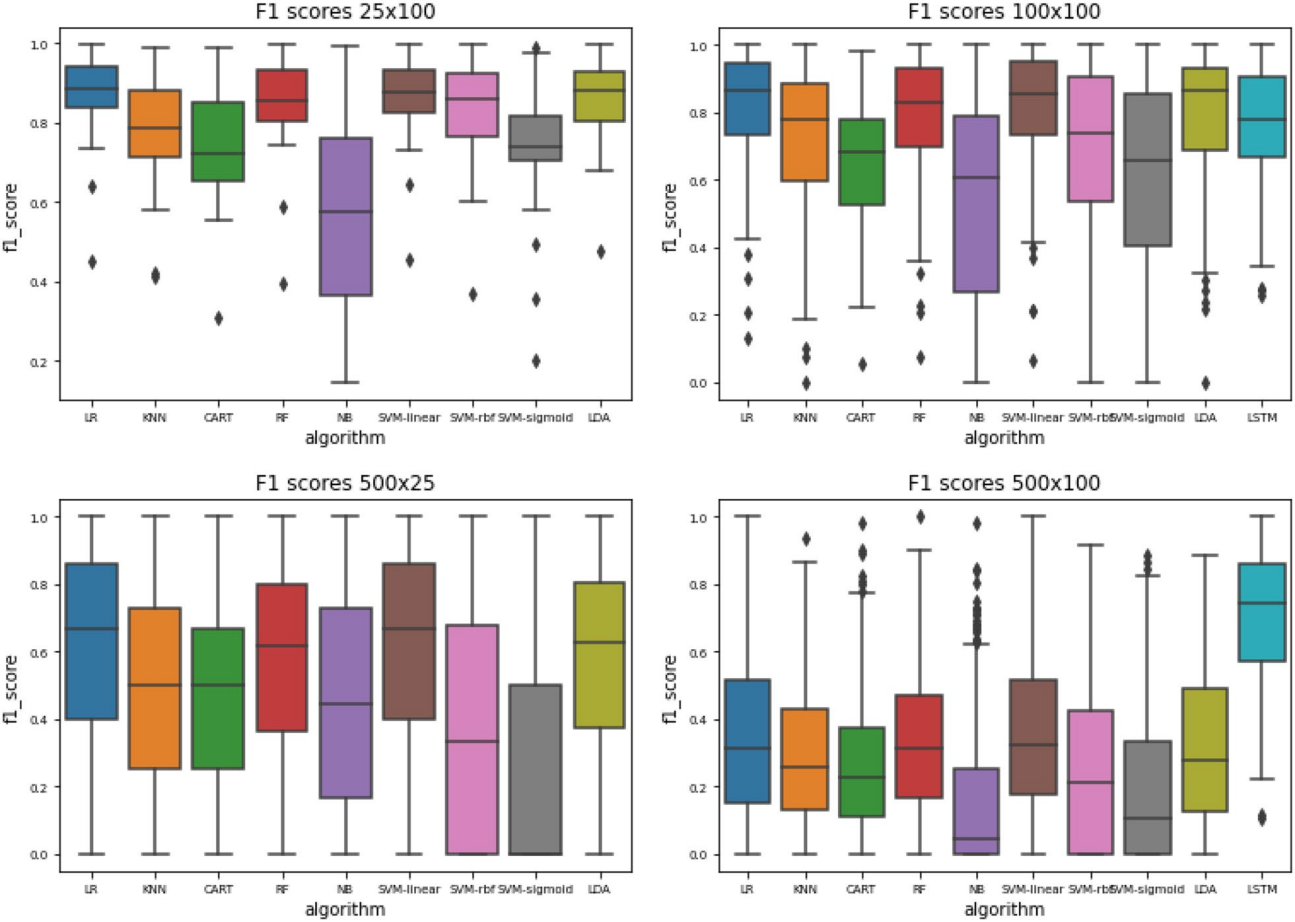
F1 scores per algorithm for the 25x100 problem, 100x100 problem, 500x25 problem and 500x100 problem. LR = Logistic regression;KNN = K-nearest neighbours; CART = Decision tree; RF = Random forest; NB = Naive Bayes; SVM = Support vector machine; LDA = Linear discriminant analysis; LSTM = Long short-term memory network

Among the classical classifiers, we see that logistic regression robustly performs best (Fig. 7), followed by linear discriminant analysis and random forests performing about the same.

Naive Bayes shows the worst performance among the classical classifiers for this problem. The large difference in performance between Naive Bayes and LDA implies that there are considerable co-variances among the fingerprint features–which would be expected–and can be confirmed by a correlation analysis. These may originate from the fact that the random walks that produce the fingerprints are performed on the same substructure, or by the way the hashes are calculated. Decision trees seem to over-fit–especially with larger sample sizes. The random forests mitigate this to some extent, but a decline in decision tree performance impacts the random forests as well.

The performance of Logistic Regression and Support Vector Machines is almost identical, which is to be expected as they use essentially the same classification method. The different loss functions used in the respective gradient descents do not have any significant impact. However, non-linear kernels had a highly negative impact on the SVM classification performance.

**Fig. 8:**
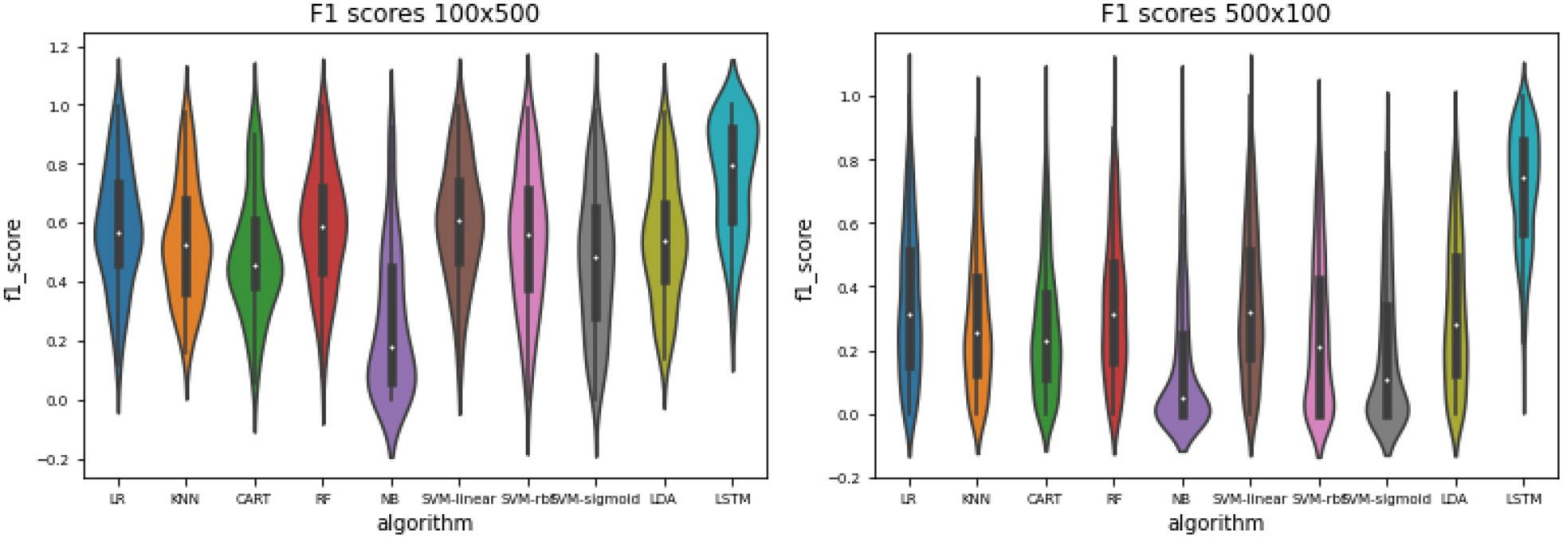
Violin plot of F1 scores per algorithm for the 100x500 problem (left) and the 500x100 problem (right). LR = Logistic regression;KNN = K-nearest neighbours; CART = Decision tree; RF = Random forest; NB = Naive Bayes; SVM = Support vector machine; LDA = Linear discriminant analysis; LSTM-Long short-term memory network

While a direct comparison should be interpreted with caution, as the LSTM is performing a different classification task to the other classifiers (i.e. multi-label rather than single-label), nevertheless, we can make some observations about the resulting F1 scores. Interestingly, we see that from the overall performance perspective, although the LSTM does not outperform the other approaches at problem size 100x100 (Fig. 7), it performs somewhat better at the 100x500 problem size, and significantly better than the other approaches for the 500x100 problem size (Fig. 8). This implies that the LSTM is, at least on the face of it, better able to scale towards the scope of the full ontology than the classical approaches, although we did not attempt to use the LSTM for problem size categories involving small numbers of members per class (e.g. with 25 members), as network performance decreased with decreased numbers of members per class, as would be expected for this type of approach.

In the remainder of this section we give more detail about the evaluation of the networks.

**Fig. 9:**
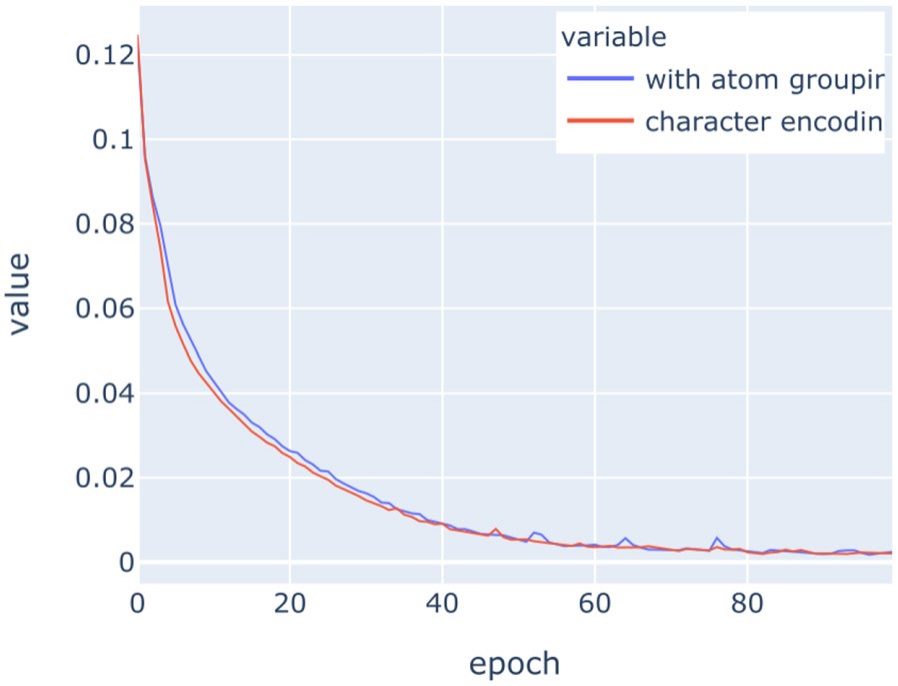
Loss on training data

**Fig. 10:**
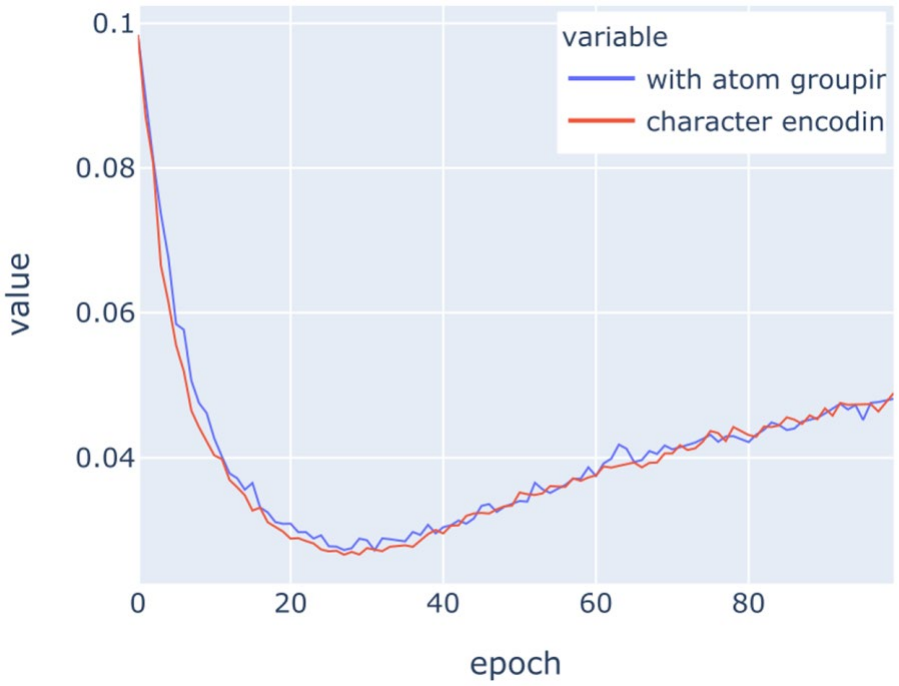
Loss on validation data

**Fig. 11:**
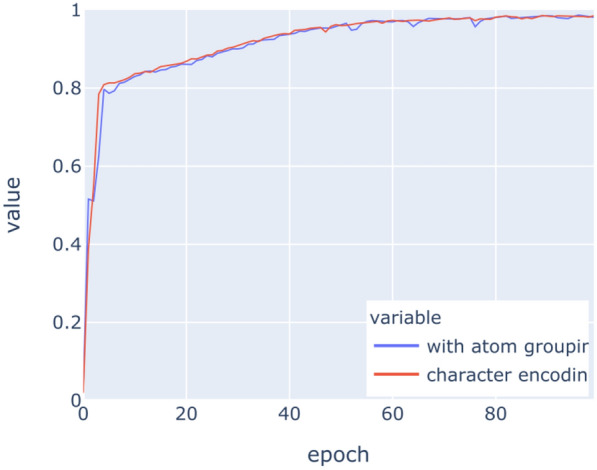
Precision on training data

**Fig. 12:**
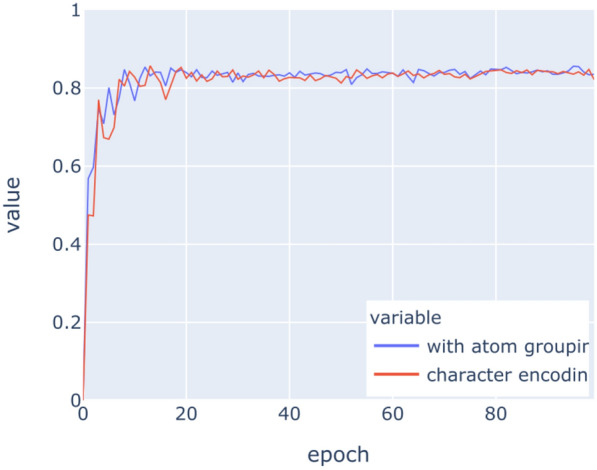
Precision on validation data

**Fig. 13:**
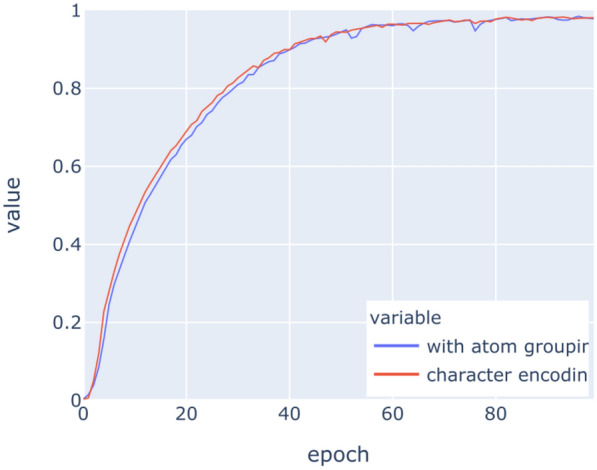
Recall on training data

**Fig. 14:**
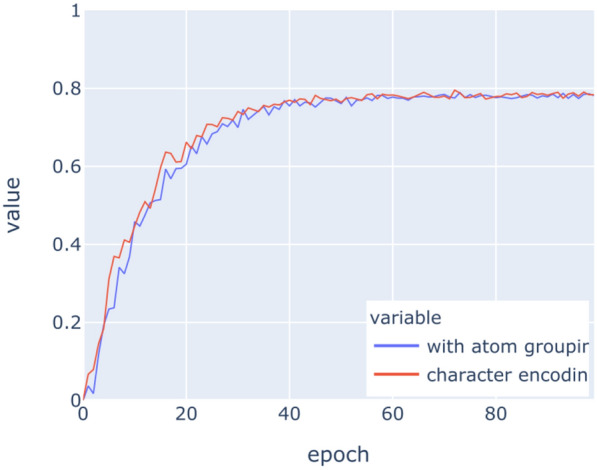
Recall on validation data

The LSTM networks have been trained on the above datasets for 100 epochs with binary crossentropy as loss function. Figures 9, 10, 11, 12, 13, 14 show the progress of different metrics during this process. The loss on the validation set rebounds after the 25th epoch, which indicates overfitting on the dataset. Surprisingly, this does not impact the precision and recall negatively. For computing precision and recall, we used a threshold of 0.5 to distinguish class membership from non-membership. Further inspection of the predictions reveals that the mentioned lack of impact is caused by the predictions diverging from the optimal answers towards the threshold, but not passing it. This means that after the turn, prediction strength decreases, since distance form the threshold can be seen as confidence about the prediction. The slight, but constant rise of precision and recall after that turn indicate an additional improvement of those, but apparently at the cost of overfitting.

The encoding of chemicals does not have any significant impact on the success of the learning task. This implies that the networks successfully learn the structure of atom labelling in SMILES strings relatively early on without much effort. Similar experiments in natural language processing [44] have been conducted and results implied that aggregations of syntactic structures have an impact on the training. Our results indicate that this impact does not exist with the aggregation of characters into atoms.

**Fig. 15:**
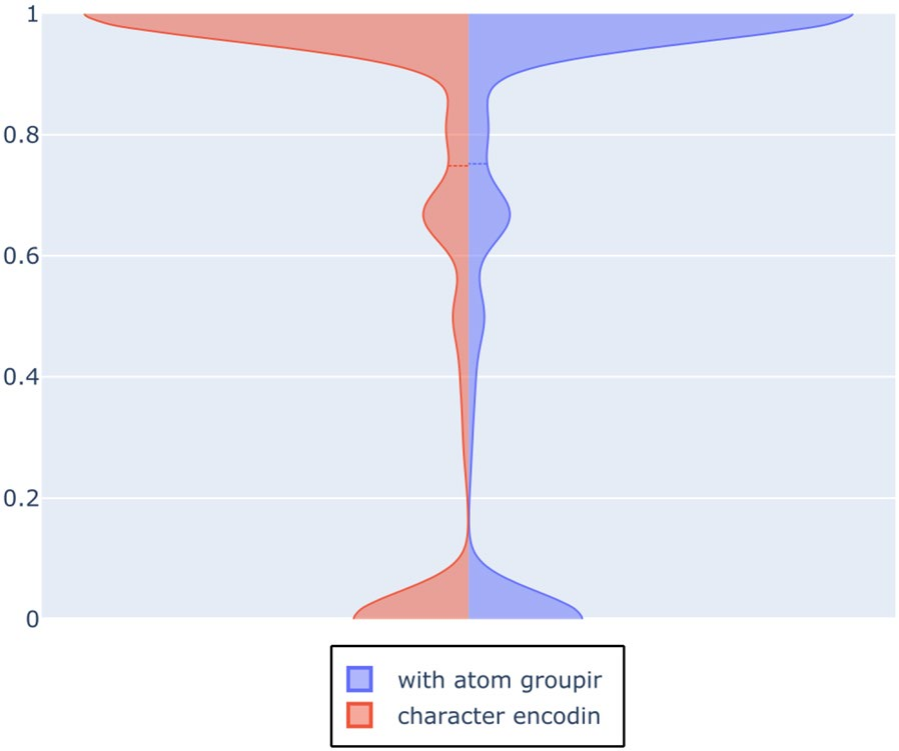
Violin plot of F1 scores on molecules in test set after 100 epochs

The violin plot in Fig. 15 depicts the distribution of F1-scores *per molecule* amongst the different molecules in the evaluation set. Note the accumulations at the ends of the scales. This shape corresponds to the response behaviour of the network. There is a subset of molecules for which the network does not give any any positive response for any class, i.e. the LSTM does not recognise the molecule as a member of any class. This behaviour is rather detrimental to the overall recall, but it is easily identifiable and thus may be used as an indicator that other approaches should be used on these molecules.

LSTMs were the approach that did not suffer greatly from larger sample sizes. One aspect of this is that the larger sample sizes create problem spaces that are more uneven, which the LSTM is better suited to handle, as the LSTM is able to make a multi-label prediction and predict multiple classes simultaneously, rather than (as is the case for the other approaches) making just a single prediction. Furthermore, as described above, the data sampling procedure from the ontology will lead to more generic classes if the number of members is larger. This implies that smaller substructures are relevant for the classification, which may be distributed widely across the actual molecules. A random walk has a lower probability of covering all the relevant aspects in this case. The LSTM consumes the whole SMILES string, which allows a more consistent classification.

Figure 8 shows that there is a large variance in performance w.r.t different chemical classes. A more class-focused analysis of the results is done in the following section.

It should be noted that we explored several configurations of LSTMs, and none of them performed better than the given configuration, whilst a substantial number showed almost identical results on the validation set. The introduction of a dropout led to a clear rise in performance, whilst different LSTM sizes and structures–even bidirectional ones–showed no positive impact. A possible reason is that there are some SMILES structures that LSTMs struggle to learn, and in future work we will explore alternative encodings to circumvent such limitations.

### By chemical class within the ontology

As can be seen by the wide distribution of F1-scores for the performances within each of the different problem sizes and algorithmic approaches, there is variance in the performance of learning for different ontology classes. At the same time, we see variance in the performance for different molecules. This prompts us to ask whether there are some general observations that we can derive about the problem of structure-based chemical ontology classification from these experiments.

**Fig. 16:**
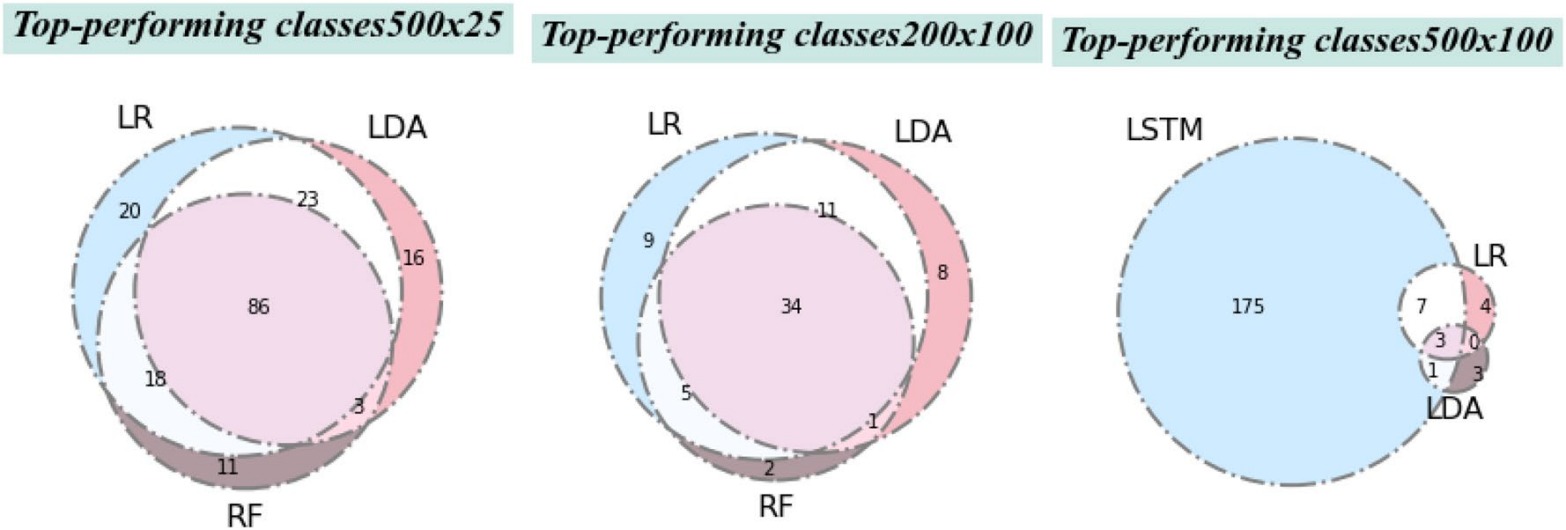
The Venn diagrams show the overlap of classes which scored F1 more than 0.8 (i.e., best-performing classes) for three of the classifiers in each of these problem sizes. LR = logistic regression; RF = random forest; LDA = linear discriminant analysis; LSTM = long short-term memory

Firstly, we can ask whether different algorithms give the same best-performing classes or different best-performing classes. Figure 16 shows the overlap of top-performing classes for different problem sizes for the three best-performing algorithms.

Figure 16 indicates that while there is a shared common core, different classifiers give partially non-overlapping sets of ‘best scoring’ classes. That is, they have partially distinct profiles with respect to the classes for which they give the best performance. This suggests that the general problem of structure-based chemical ontology classification might benefit from ensemble-based approaches that integrate results across different approaches.

In general, the classical classifiers perform best on classes that have relatively few members and well-defined structural features. For example, the best performers (F1 = 1.0) using the LR algorithm all have fewer than 50 members (Table 1).

**Table 1:** Highest and lowest-scoring classes using the LR algorithm

Class_id	f1_score	Class_name	Class_members
CHEBI:61689	1.0	Amino cyclitol	41
CHEBI:26253	1.0	Polyprenylhydroquinone	45
CHEBI:134209	1.0	Aporphine alkaloid	54
CHEBI:132157	1.0	Hydroxy-1,4-naphthoquinone	35
CHEBI:17810	1.0	1-O-(alk-1-enyl)-2-O-acyl-sn-glycero-3-phospho...	46
CHEBI:17636	1.0	Sphingomyelin d18:1	46
CHEBI:26255	1.0	Prenylquinone	38
CHEBI:83563	1.0	Long-chain alkane	29
CHEBI:60687	1.0	Cembrane diterpenoid	35
CHEBI:38836	1.0	1-benzothiophenes	43
CHEBI:36685	1.0	Chlorocarboxylic acid	33
CHEBI:75946	1.0	Cytochalasan alkaloid	30
CHEBI:38768	1.0	Phthalazines	41
CHEBI:80291	1.0	Aliphatic nitrile	35
CHEBI:38769	1.0	Indazoles	45
CHEBI:37531	1.0	Polyprenyl diphosphate	41
CHEBI:58168	1.0	1-O-acyl-sn-glycero-3-phosphocholine	37
CHEBI:83876	1.0	Cationic sphingoid	30
CHEBI:64590	1.0	Monoalkyl-sn-glycero-3-phosphocholine	30
CHEBI:131903	1.0	Pyranopyrazole	32

Class_id	f1_score	Class_name	Class_members
CHEBI:16733	0.0	D-alpha-amino acid	51
CHEBI:48544	0.0	Methanesulfonates	54
CHEBI:33702	0.0	Polyatomic cation	2178
CHEBI:47704	0.0	Ammonium salt	38
CHEBI:59869	0.0	L-alpha-amino acid zwitterion	53
CHEBI:50128	0.0	Biflavonoid	53
CHEBI:25414	0.0	Monoatomic monocation	32
CHEBI:46899	0.0	Benzothiazine	37
CHEBI:35218	0.0	Anthocyanin cation	47
CHEBI:64985	0.0	Bioconjugate	39
CHEBI:33639	0.0	Ortho- and peri-fused compound	56
CHEBI:38716	0.0	Carboxylic acid dianion	311
CHEBI:59635	0.0	Organophosphonate oxoanion	38
CHEBI:35284	0.0	Ammonium betaine	1205
CHEBI:29089	0.0	1,2-diacyl-sn-glycerol 3-phosphate	52
CHEBI:35296	0.0	Ortho-fused polycyclic arene	38
CHEBI:26469	0.0	Quaternary nitrogen compound	1317
CHEBI:38037	0.0	Methanesulfonate salt	40
CHEBI:76176	0.0	2-hydroxy fatty acid anion	43
CHEBI:59558	0.0	Medium-chain fatty acid anion	36

The worst-performing classes for the classical classifiers, exemplified by the worst-ranked for the LR algorithm indicated in Table 1, include those with features not directly represented in the fingerprint, such as D-stereochemistry, cations and salts. The information that would be required to make these classifications is just not available for these classifiers to learn, however, these could be improved with the adoption of alternative fingerprinting strategies.

The profile of poor performers is different for the LSTMs compared to the classical approaches (Table 2).

**Table 2:** Highest and lowest-scoring classes using the LSTM algorithm

Class_id	f1_score	Class_name	Class_members
CHEBI:17984	1.000000	Acyl-CoA	696
CHEBI:37240	0.998350	Adenosine 3’,5’-bisphosphate	697
CHEBI:22251	0.995114	Adenosine bisphosphate	702
CHEBI:61078	0.993104	Purine nucleoside bisphosphate	706
CHEBI:58946	0.992382	Acyl-CoA oxoanion	707
CHEBI:61079	0.991522	Ribonucleoside bisphosphate	707
CHEBI:51277	0.990164	Thioester	745
CHEBI:37123	0.989925	Nucleoside bisphosphate	708
CHEBI:60971	0.989796	Aminophospholipid	104
CHEBI:18303	0.989796	Phosphatidyl-L-serine	104
CHEBI:64583	0.986667	Sphingomyelin	251
CHEBI:58342	0.985302	Acyl-CoA(4-)	613
CHEBI:74927	0.985222	Furopyran	934
CHEBI:35766	0.983871	Glycerophosphoserine	129
CHEBI:78799	0.983607	Hydroxy fatty acid ascaroside	152
CHEBI:52565	0.980769	Acylglycerophosphoserine	114
CHEBI:26875	0.980392	Terpenyl phosphate	133
CHEBI:36233	0.980392	Disaccharide	156
CHEBI:64482	0.979315	Phosphatidylcholine	623
CHEBI:57643	0.979315	1,2-diacyl-sn-glycero-3-phosphocholine	621

Class_id	f1_score	Class_name	Class_members
CHEBI:64365	0.333333	Aralkylamino compound	138
CHEBI:22715	0.333333	Benzimidazoles	352
CHEBI:23697	0.328358	Dichlorobenzene	452
CHEBI:48470	0.322581	Amidobenzoic acid	148
CHEBI:25235	0.320000	Monomethoxybenzene	247
CHEBI:46848	0.315789	N-arylpiperazine	176
CHEBI:51681	0.305085	Dimethoxybenzene	269
CHEBI:26455	0.294118	Pyrroles	200
CHEBI:83403	0.293333	Monochlorobenzenes	429
CHEBI:50995	0.292683	Secondary amino compound	417
CHEBI:27024	0.277778	Toluenes	135
CHEBI:37407	0.259887	Cyclic ether	806
CHEBI:73539	0.256410	Naphthyridine derivative	162
CHEBI:26878	0.251429	Tertiary alcohol	756
CHEBI:36786	0.246246	Tetralins	108
CHEBI:27116	0.235294	Trihydroxyflavone	150
CHEBI:38338	0.235294	Aminopyrimidine	149
CHEBI:33572	0.222222	Resorcinols	153
CHEBI:83812	0.114286	Non-proteinogenic amino acid derivative	145
CHEBI:13248	0.108108	Anilide	279

The best-performing classes with the LSTM approach also include classes that have well-defined structural features, but these have far more members than the best performers in the LR approach, illustrating the ability of the LSTM to cope with larger problem sizes–and the added value of additional examples to learn from.

The worst-performing classes for the LSTMs have a quite different profile to those of the LRs, and as expected do not include salt or ion classes. Rather, somewhat intriguingly, we see many examples of classes with complex ring structures, especially aromatic or substituted ring structures.

**Fig. 17:**
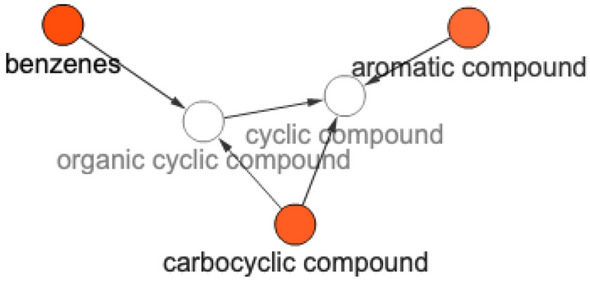
Enrichment analysis result on the ChEBI structural ontology for the 50 worst-performing classes in the LSTM

To confirm this observation, we applied the BiNChE chemical enrichment analysis utility [16] on the 50 worst-performing classes from the LSTM-only set. We see a number of clear enrichments–benzenes, aromatic compounds, and carbocyclic compounds (Fig. 17), while in the worst-performing classes from all algorithms we see no similar enrichment.

We can hypothesise that the poor performance for the aromatic molecules with the LSTM may be due to the fact that aromaticity can be encoded in SMILES strings in multiple different ways–using alternating single and double bonds, or using lowercase letters. It is plausible that the network did not learn that e.g. the aromatic ‘c’ carbon atom is in fact the same atom type as the typical ‘C’ in another molecular representation, and treated them as different entities. Larger datasets from possibly synthetic sources or a more homogenous representation of aromatic components may help the network to learn those abstractions. It is also worth observing that in general the LSTM can be expected to have more difficulty with parsing cycles from SMILES strings than linear molecular structures because these structures are broken up during the translation of a molecule into its SMILES representation.

### Comparison to the state of the art

As a final evaluation, we compare our results to the state of the art structure-based ontology classification tool, ClassyFire [3]. We do this comparison using as input the 500x100 problem size dataset, by executing ClassyFire on the SMILES strings associated with the test set of molecules, encompassing 20% of the full 50,000 set of molecules, i.e. 10,000 sample molecules with SMILES. Of these, ClassyFire was unable to process 501 of them due to errors in the generation of an InChI (IUPAC international chemical identifier, [45]) from the SMILES. ClassyFire uses an InChI-Key-indexed cache of parent classes for molecules that have been previously classified in order to speed up its classification performance, as matching multiple substructural patterns is expensive. There are known to be certain molecules for which it is not possible to generate an InChI but for which it is possible to have a SMILES, and these 501 molecules are of this type–mainly due to the explicit representation of attachment points within the SMILES, e.g. the following SMILES: ‘C(C(COP(=O)(OC[C@@H](C(=O)O)N)O)OC(=O)*)OC(=O)*’. There were also a few entries for which ClassyFire returned other errors. In total, we received 9,484 classification results for our 10,000 sample molecules. Each classification result includes multiple ChEBI classes including the very high-level ChEBI classes such as ‘molecular entity’. We condensed these to only include classes that were not superclasses of each other.

**Fig. 18:**
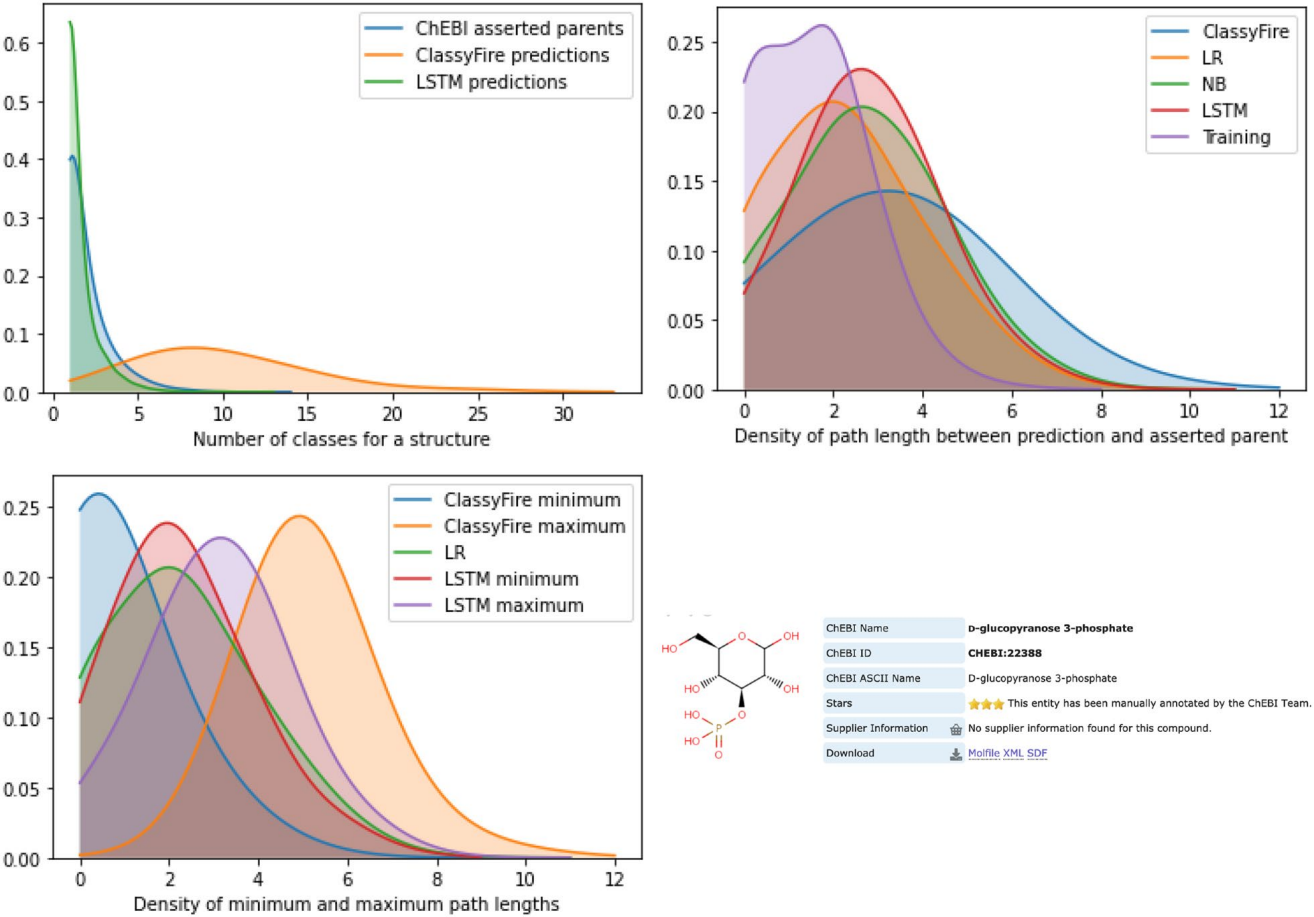
**a** Number of parent classes in different approaches. **b** Path length to asserted parent class in different approaches. **c** Minimum and maximum path lengths to asserted parent classes. **d** D-glucopyranose 3-phosphate, an example molecule for which ClassyFire performs poorly on this metric. *Training* refers to the selection of members for classes in the learning task

It is not straightforward to make a direct comparison between our results and the performance of the ClassyFire tool, for various reasons. First, ClassyFire uses a different underlying ontology to ChEBI that is only partially mapped to ChEBI. The ontologies differ in some fundamental ways in their treatment of chemical classes. For example, ClassyFire’s classes include molecules with different charge states, encompassing conjugate bases and acids in the same grouping, while ChEBI strictly separates these. Therefore, ChEBI class predictions returned by ClassyFire may be less precise than the ClassyFire original class. However, our dataset is restricted to the ChEBI classification from which it was generated. Second, ClassyFire makes multiple parent class predictions, while our classical classifiers make only a single best match parent class prediction, and although the LSTM is able to make multiple predictions, it makes far fewer predictions than ClassyFire does. Figure 18a shows a kernel density diagram for the number of parent classes in different approaches: (1) ChEBI directly asserted parents (with a mean of 1.816 parent classes per leaf structure across the full ontology), (2) the LSTM predicted parent classes (mean = 1.435 in the 500 x 100 problem), and (3) ClassyFire predicted parent classes (mean = 9.926). For both ClassyFire and the LSTM, these counts exclude any parent classes returned by the algorithm that are superclasses of any of the other parent classes. Finally and most importantly, ClassyFire has since its initial release in 2016 been used in the development of ChEBI: it is used in the bulk submissions pipeline to automatically classify entities that are incorporated into ChEBI before they can be manually curated. This means that ClassyFire has actually produced a portion of the classifications in our dataset (both training and test), although these are not flagged or indicated as such in any way. This introduces a bias which it is difficult to fully address.

We compare the approaches by computing a path length distance between what we might call the ‘ground truth’ of the asserted classification in ChEBI, and the predicted classification. That is, we count the number of subclass relations that must be traversed to get from a directly asserted parent to the predicted parent. In practice, the longer paths tend to reflect classifications that are either wrong (in a different ontology branch) or not very useful (as very high level). Thus, path length provides a useful metric for the quality of a classification. As there may be multiple directly asserted parents and multiple predicted parents, for each structure in the test set, we computed all path lengths between pairwise combinations of asserted and predicted classes. If the predicted class was identical with one of the asserted parents, we added a path length of 0 to indicate a match. Note that the asserted parents in ChEBI are not always the class which we used as input to our classifiers, due to the selection processing of the ontology for learning purposes. Thus, we compute the path lengths also on the classes that we used as the selection. Figure 18b illustrates the overall density of the returned path lengths in this metric, with the selected classes indicated as ‘Training’, showing the results for the LSTM, the best and worst of the classical approaches, and ClassyFire. It can be seen that ClassyFire returns the widest range of path lengths on this metric with a mean path length of 3.20, while the LR (mean = 2.29) outperforms the NB (mean = 2.74) and the LSTM (mean = 2.81) which appear to perform similarly. The training baseline for our learning approaches has mean = 1.48.

These results may reflect a bias based only on the number of paths computed. For that reason we calculated also the minimum path length and the maximum path length (Fig. 18c). On the minimum path length, ClassyFire outperforms the other approaches, while on the maximum path length it shows the worst performance. However, in practice for a novel structure it would not be known without manual inspection which of the results returned was the best classification–reducing the benefit of using an automated approach. While ClassyFire provides an ordering in the class list for their own ontology that can be used to prioritise classification, they do not provide a similar ranking in their prediction of ChEBI terms. To illustrate why the maximum path length of ClassyFire is significantly longer than for the other approaches, we consider the molecule ‘D-glucopyranose 3-phosphate’ as an example. In ChEBI it is classified as a glucose phosphate that is a derivative of hexose. ClassyFire returns the following predicted classifications for this molecule: ‘primary alcohol’ (CHEBI:15734), ‘secondary alcohol’ (CHEBI:35681), ‘ether’ (CHEBI:25698), ‘monoalkyl phosphate’ (CHEBI:25381), ‘polyol’ (CHEBI:26191), ‘oxanes’ (CHEBI:46942), ‘hemiacetal’ (CHEBI:5653), ‘hexose’ (CHEBI:18133), and ‘organic oxide’ (CHEBI:25701). Many of these classifications relate to correct but very general chemical groupings, illustrating the challenges with the substructure-based approach to automated structure-based ontology classification in the context of the large and combinatorial chemical structural landscape. Other classifications are incorrect in ChEBI due to differences between ClassyFire and ChEBI’s approach to classification (e.g. hexose vs. hexose derivative).

ClassyFire has already had enormous impact on the fields of chemical data management and -omics dataset analysis by enabling novel structures to be classified. However, our results underline that there is a role for dynamic machine learning-based approaches alongside substructure-based approaches. ClassyFire predicts for each molecule significantly more (non-redundant) parent classes (mean = 9.926) than the LSTM (mean = 1.435) or the LR (1 prediction), where—roughly speaking—often, one of these predicted parent classes is better than the classes predicted by the LSTM or the LR in our path distance metric (bearing in mind the possible bias due to the use of ClassyFire in ChEBI development), but most of them are worse. ClassyFire seems particularly suitable for semi-automatic use cases, where the results that are returned by ClassyFire are validated manually. However, if manual validation is not possible, the other approaches appear to be more suitable, in particular if they can be used together in a way that plays to the differential strengths of the different approaches. Importantly, they are also likely to be easier to maintain and extend going forward.

## Conclusions and future work

Our objective was to evaluate the applicability of machine learning approaches to the problem of structure-based chemical ontology classification, and we can conclude that indeed machine learning seems a very promising approach for this complex problem.

There are a few final observations we can make about our investigation. Firstly, while for the overall problem the LSTM is clearly the best-performing approach due to the advantages it has of dealing appropriately with the embedded ontology class structure, and the dynamic potentials of the variable-length input, it struggles with classes that have only a small number of members to learn from. Moreover, there was a subset of molecules for which the LSTMs gave no class predictions at all, and the LSTM also showed specific weaknesses in terms of smaller molecules and aromatic classes. On the other hand, for classes that have relatively small numbers of members (i.e. more specific classes) and which are non-overlapping, logistic regression outperformed all other approaches while being fast and straightforward, and random forests also achieved good performance while having the added potential benefit of explainability. No single approach gives the best results in all the problem cases, and different approaches give partially non-overlapping sets of ‘best scoring’ classes, that is, they have partially distinct profiles with respect to which classes they perform best for. This is interesting because it implies that a hybrid approach may have promise for solving the problem of structure-based chemical ontology classification more generally than an approach based on a single classifier, and further research is needed to determine how to harness the best elements of the different approaches into a single unified system.

There are several parameters of the overall problem that we did not address yet, and which we leave for our future research. We did not yet attempt any dimensionality reduction on the data in advance of the learning, which might boost scalability and performance. We also did not try to evaluate different fingerprints, for example circular fingerprints or fingerprints including explicit ring systems, which we could anticipate would achieve better performance by making more information available to the classifiers. We also plan to explore the use of an enhanced molecular structure representation such as DeepSMILES [46], which was developed for the problem of generative neural network-based structure generation, to determine whether this will improve the predictions in the areas that the LSTM is currently weak.

There also remain several additional challenges in extending our current approach to be able to classify molecules into a full chemical ontology at the scale of ChEBI, rather than the artificially selected subsets of the ontology data that we have used thus far. The shape of the ChEBI ontology means that the full prediction task is in fact a *sparse* multi-label classification problem, as while each molecule belongs to multiple classes, nevertheless for each molecule only a few classes are assigned relative to the (large) overall ontology. Even when considering all possible classes by traversing the full ontology, the class vectors for each molecule are sparse. Taken together with the fact that class sizes are not in fact balanced, this makes the classification task more difficult. Therefore, as a follow-up investigation, we intend to consider weight balancing in our regression and SVM models and a modification in the loss function of our deep learning models.

*Self-attention* has recently been shown to be a central contributing factor when accounting for long-term dependencies among different tokens [47]. While using SMILES strings is practical, considering the molecular graph *as a graph* is an alternative structural representation as input for the classification task. Currently, an active research topic in the field of language modeling is to examine if the meaning of a sentence can be inferred by combining the meanings of words to determine the meaning of larger units, i.e., to learn a composition function that can be applied on smaller constituents to give a representation for bigger semantic units [48, 49]. An extension of our approach would thus be to incorporate the compositional structure of the chemical compounds by using tree-structured Recursive Neural Networks (RvNNs). Different non-linear network structures may also improve this point. Graph neural networks [50] traverse the graph structures to find transformation-invariant features that are then used as inputs for the classification task. Neural networks based on Latent Compositional Representations [51] may be used to get better insight on the actual substructures that classifications have been based on, while preserving this information along the whole processing chain.

Finally, the chemistry domain includes broad regularities that can be axiomatised in logical languages [2], especially at the higher hierarchical levels within the chemical ontology, such as for the definitions of *molecules*, *ions* and *salts*, or representing disjointness between *organic* and *inorganic* entities, and so on. These distinctions are only partially encoded at present in the weakly axiomatised ChEBI ontology through the use of relationships such as *has part*, but they can be supplemented by extensions both within OWL (e.g. [52]) and in higher order logics (e.g. [53]) to more formally capture the logical rules of chemical classification. Neuro-symbolic learning approaches encode logical axioms in ways that can be used by ANNs in order to derive systems that can learn both from facts and axioms [54, 55], thus potentially improving performance while simultaneously reducing the amount of data that is needed for training. These systems might be particularly potent for chemical ontology classification if ChEBI were to be enhanced with a stronger axiomatisation, allowing neural networks to be built that improve the classification performance whilst remaining interpretable and based on expert knowledge. Another possible neuro-symbolic approach would be to use hierarchical classification based on an ontology for which the immediate subclasses of a given parent class provides a disjoint partition. The class structure in ChEBI does not satisfy this requirement. Hence, one would need to create an ontology using feature-based classes in the style of concept lattices [56], and embed ChEBI into this ontology. We plan to explore in the future whether such approaches can yield a benefit for structure-based chemical ontology classification.

## Data Availability

Source code is available on GitHub: https://github.com/MGlauer/cheleary and https://github.com/jannahastings/chebiutils. The study uses data from the ChEBI database which is freely available at http://www.ebi.ac.uk/chebi. All data that has been used in the experiments and the respective results are available at https://doi.org/10.5281/zenodo.4519815.
